# Onion *(Allium cepa)* extract prevents cadmium induced renal dysfunction

**DOI:** 10.4103/0971-4065.59334

**Published:** 2009-10

**Authors:** S. F. Ige, E. O. Salawu, S. B. Olaleye, O. A. Adeeyo, J. Badmus, A. A. Adeleke

**Affiliations:** Department of Physiology, Faculty of Basic Medical Sciences, Ladoke Akintola University of Technology, Ogbomoso, Oyo State, Nigeria; 1 Department of Physiology, Faculty of Medicine, University of Ibadan, Ibadan, Oyo State, Nigeria; 2 Department of Anatomy, Faculty of Basic Medical Sciences, Ladoke Akintola University of Technology, Ogbomoso, Oyo State, Nigeria; 3 Department of Biochemistry, Faculty of Basic Medical Sciences, Ladoke Akintola University of Technology, Ogbomoso, Oyo State, Nigeria

**Keywords:** *Allium cepa*, cadmium, heavy metals, renal clearance, reactive oxygen species

## Abstract

Cadmium (Cd), a heavy metal, is known for its adverse effects on the body. In this study, the lowering effect of Cd on renal clearance (RC) was investigated, and *Allium cepa* extract (AcE) (an antioxidant) was pre-administered orally to prevent Cd's adverse effects. Seventy-two Wistar rats, grouped into three (n = 24), were used for this study. While Group C was given 1.0 ml of AcE daily (orally), Group A and Group B were given distilled water. AcE administration was done for eight weeks. Afterwards B and C were then given 1.5 ml/kg BW of 0.3 mg/L 3CdSO_4_.8H_2_O intraperitoneally for three consecutive days. The results obtained showed that Cd causes significant reduction in the 24 hour urine volume (from 3.017 ± 0.125 to 2.433 ± 0.118 ml), RC (from 3.258 ± 0.114 to 1.357 ± 0.104 ml/h for creatinine; and from 0.350 ± 0.057 to 0.185 ± 0.055 ml/h for urea), plasma and tissue SOD and CAT activity (form 1.644 ± 0.036 to 1.307 ± 0.056 u/g protein for plasma SOD; 0.391 ± 0.029 to 0.2692 ± 0.031 u/protein for plasma CAT; 1.695 ± 0.034 to 1.327 ± 0.049 u/g protein for tissues SOD; and from 0.350 ± 0.027 to 0.273 ± 0.043 u for tissue CAT), and significant MDA increased in plasma (from 1496.79 ± 1.321 to 1679.48 ± 143.29 μg/g protein) and tissue (from 1265.22 ± 2.285 to 1669.87 ± 14.61 μg/dL). AcE, however, prevents these Cd's adverse effects. This findings lead to the conclusion Cd exposure causes renal dysfunction, but oral administration of onion could prevent it.

## Introduction

Our environment and diet expose us to cadmium (CD).[1] Although Cd exposure may contribute to essential hypertension,[2] affect respiratory system, cause bone diseases, and affect reproductive system, the kidney remains the critical target organ for cadmium toxicity.[3,4] Most studies on cadmium toxicity, therefore, centres on the detection of early signs of kidney dysfunction which results from oxidation.[5] In other words, Cd causes its injurious effects by generation of reactive oxygen species (ROS).[5]

On the contrary, *Allium cepa* (containing sulphur compounds and flavonoids) has anti-oxidant properties.[6] This research work was, therefore, targeted at knowing whether or not oral administration of the *AcE* will prevent cadmium's adverse effects of on renal clearance as a representative of renal functions.

## Materials and Methods

A total of 72 adult male Wista rats (180-220 g) were used for this study. They were inbreed at the Animal House section of the Department of Physiology, Ladoke Akintola University of Technology, Ogbomoso, Nigeria. The animals were acclimatized over a period of two weeks.

### Preparation of *Allium cepa* extract

*AcE* was prepared following a method described by Nelson *et al*.[7] Fresh *Allium cepa* bulbs were rinsed thoroughly in distilled water and air dried; 200 grams were then blended. The resulting paste was allowed to stand for 24 hours. Juice was then filtrated and squeezed out of it. The extract was stored bellow 4°C.

### Grouping of animals and treatment

The rats were grouped into three groups (n = 24). Group A served as the control and was given distilled water for 10 weeks followed by intraperitoneal injection of normal saline. Animals in Group B were also given distilled water for the eight weeks but followed by intraperitoneal administration of cadmium (in the form 1.5 ml/kg BW of 0.3 mg/L of 3CdSO_4_.8H_2_O). Group C animals were treated with 1.0 ml of AcE/day for 10 weeks and afterwards administered cadmium (in the form 1.5 ml/kg BW of 0.3 mg/L of 3CdSO_4_.8H_2_O) intraperitoneally. Administration of cadmium was for three consecutive days.

### Animal sacrifice and collection of samples

Twenty-four hours after the last intra-peritoneal injection of cadmium, each animal was weighed and then transferred into metabolic cage equipped with accessory for collecting urine. The 24 hr urine sample was collected and its volume recorded for each rat. Each rat was then sacrificed by cervical dislocation and blood samples were collected via cardiac puncture. Blood sample obtained from each rat was divided into two: one half in plain bottle, and the other half in EDTA bottle. Plasma and serum were obtained by centrifugation at 3,000 rpm for 20 minutes.

### Collection of data and statistical analysis

The weight increase (g) and 24 hr urine volume (ml) were recorded. Urine and serum creatinine concentration were determined using alkaline picrate method described by Jaffe (1886).[8] Urine and serum urea concentration were determined using diacetylmonoxime method described by Ceriotti *et al*. (1963).[9] Renal clearance was then calculated using the formula “Clearance of Y = (Urine conc. of Y × 24 hr Urine volume)/Plasma conc. of Y” as documented by Guyton and Hall (2001).[10] Plasma and tissue superoxide dismutase (SOD) activity were determined using the method described by Fridovich (1989).[11] Plasma and tissue catalase (CAT) activity were determined using the method described by Sinha (1972).[12] Plasma and tissue malondialdehyde (MDA) Concentrations were determined using the procedure described by Varshney and Kale (1990).[13]

The data obtained are presented as mean ± SD. The “Control Group” and the “Test Groups” were compared using the Mann-Whitney *U*-test. The significance level was set to a *P* < 0.05.

## Results

The following results were obtained and are presented as mean ± SEM. Level of significance is taken at *P* < 0.05.

### Weight increase

There was significant weight gain in all the groups over the period of the research. There was, however, no significant difference in weight gain of Group B and Group C relative to the control.

### Kidney weight

The kidney weight of Group B as well as that of Group C was not significantly (*P* > 0.05) different from that of the control.

### Twenty four (24) hour urine volume

The 24 Hr urine volume was found to be significantly (*P* < 0.05) lower for Group B with respect to the control. The 24 Hr urine volume for Group C was, however, not significantly (*P* > 0.05) different from that of the control.

### Creatinine clearance

Creatinine clearance was significantly (*P* < 0.01) lower in Group B compared to the control. Creatinine clearance of Group C, on the contrary, was not significantly different from that of the control.

### Urea clearance

Urea clearance was also found to be significantly (*P* < 0.05) lower in Group B compared to the control. Urea clearance of Group C, on the contrary, was not significantly different from that of the control.

### Plasma superoxide dismutase activity

Plasma SOD activity was highly significantly (*P* < 0.01) lower for Group B with respect to the control. But, plasma SOD activity of Group C was not significantly different from that of the control.

### Plasma catalase activity

Plasma CAT activity was highly significantly (*P* < 0.01) lower for Group B with respect to the control. On the contrary, Plasma CAT activity of Group C was not significantly different from that of the control.

### Plasma malondialdehyde concentration

Plasma MDA concentration was significantly (*P* < 0.05) lower for Group B with respect to the control. However, Plasma MDA concentration of Group C was not significantly different from that of the control.

### Tissue superoxide dismutase activity

Tissue SOD activity was highly significantly (*P* < 0.01) lower for Group B with respect to the control. Tissue SOD activity of Group C was, however, not significantly different from that of the control.

### Tissue catalase activity

Tissue CAT activity was highly significantly (*P* < 0.05) lower for Group B with respect to the control. On the contrary, Tissue CAT activity of Group C was not significantly different from that of the control.

### Tissue malondialdehyde concentration

Plasma MDA concentration was significantly (*P* < 0.05) lower for Group B with respect to the control. On the other hand, Plasma MDA concentration of Group C was not significantly different from that of the control.

## Discussion

The results of this study show that neither chronic administration of 1.0 ml AcE nor acute exposure to cadmium significantly affects weight increase [Table 1]; this supports the findings of Campos *et al*.[14] that acute exposure to cadmium mainly affects the respiratory system and gastrointestinal system but has no immediate effect on body weight. This can be linked to the low caloric and low protein (approximately 1% by weight) content of *Allium cepa*.[15] On the other hand cadmium did not cause any significant difference in weight gain probably because it was administered for only three days (acute exposure). Chronic administration of cadmium (for twelve weeks) in a previous study[16] showed that Cd significantly (*P* < 0.05) affects weight gain. These same reasons could be responsible for the non significant difference in the kidney weight across the three groups [Table 2].

**Table 1:** Weight increase across the three groups during the 10 weeks of research

	Group A	Group B	Group C
Weight before sacrifice (g)	211.500 ± 0.369	213.500 ± 0.520	208.333 ± 0.393
Initial weight (g)	177.667 ± 0.410	179.500 ± 0.400	178.167 ± 0.509
Weight increase (g)	33.833 ± 0.402	34.000 ± 0.491	30.167 ± 0.551
*P* (when compared with control)		0.4848	0.2426

**Table 2:** Comparison of kidney weight across the groups

	Group A	Group B	Group C
Kidney weight (g)	0.5655 ± 0.039	0.5701 ± 0.041	0.563 ± 0.042
*P* (when compared with control)		0.4457	0.0776

Table 3 shows that there was no significant difference in 24 hour urine volume of animals pretreated with onion before cadmium administration and the control, while there was significant (*P* > 0.05) reduction in animals treated with cadmium only. This goes along with the findings of Asagba and Obi[17] that cadmium toxicity brings about a reduction in 24 hour Urine Volume. This is because cadmium (as well as most other heavy metals) interferes with glomerular filtration rate (GFR) and tubular processes (tubular re-absorption and tubular secretion)[18] which are the major determinants of Urine Volume. The pre-administered onion would therefore be responsible for the prevention of these lowering effects of cadmium on 24 hour Urine volume (ml) by preventing the lowering of GFR.

In a similar way renal creatinine clearance (RCC) and renal urea clearance (RUC) of animals pretreated with onion before cadmium administration are not significantly different (*P* > 0.05) from those of the Control. Meanwhile, animals treated with cadmium showed a highly significant (*P* < 0.01) decrease in RCC [Table 4] and a significant decrease (*P* < 0.05) in RUC [Table 5] compared to the control. This supports the findings of Machiko *et al*. (1978)[19] which says that cadmium toxicity brings about reduction in renal clearance among other renal functions. Therefore, *Allium cepa* (onion) was able to prevent cadmium's adverse effects on RC which could serve as a representative of renal functions. In other words, onion is able to prevent Cd's adverse effect on renal functions.

**Table 3:** Comparison of 24 hour urine volume across the groups

	Group A	Group B	Group C
24 hr urine volume (ml)	3.017 ± 0.125	2.433 ± 0.118*	2.983 ± 0.131
*P* (when compared with control)		0.0446	0.4623

* *P* < 0.05

**Table 4:** Comparison of creatinine clearance (ml/hour) across the three groups

	Group A	Group B	Group C
Creatinine clearance	3.258 ± 0.114	1.357 ± 0.104**	3.194 ± 0.096
*P* (when compared with control)		0.0092	0.05379

** *P* < 0.01

**Table 5:** Comparison of urea clearance (ml/hour) across the three groups

	Group A	Group B	Group C
Urea clearance	0.350 ± 0.057	0.185 ± 0.055*	0.322 ± 0.053
*P* (when compared with control)		0.0150	0.0532

* *P* < 0.05

There was no significant difference in SOD activity of both the plasma and tissue of the control and that of the animals pretreated with onion before cadmium exposure. On the contrary, there was a highly significant (*P* < 0.01) decrease in plasma [Table 6] and tissue [Table 7] SOD Activity in animals treated with cadmium only compared to the control. This finding is in agreement with Jamall and Crispin (1985),[20] and is at the same time in support of *Allium cepa* as an antioxidant,[6] since increase in SOD activity is linked to reduction in the activities of reactive oxygen species.

**Table 6:** Plasma superoxide dismutase activity (/g protein) across the groups

	Group A	Group B	Group C
Plasma SOD activity	1.644 ± 0.036	1.307 ± 0.056**	1.644 ± 0.059
*P* (when compared with control)		0.0029	0.5000

** *P* < 0.001

**Table 7:** Tissue superoxide dismutase activity (/g protein) across the groups

	Group A	Group B	Group C
Tissue SOD activity	1.6952 ± 0.034	1.3270 ± 0.049**	1.6444 ± 0.055
*P* (when compared with control)		0.001	0.1567

** *P* < 0.01

Both plasma [Table 8] and tissue [Table 9] cAT activity of animals treated with cadmium only were significantly (*P* < 0.01 and *P* < 0.05, respectively) low relative to Control. There was, however no significant (*P* > 0.05) difference between the control and the animals pretreated with onion before cadmium administration in this respect. Since increase in CAT activity is, as well, linked to reduction in the activities of reactive oxygen species and vice versa, it is therefore further established that *Allium cepa* must have reduced the oxidative stress that cadmium could cause.

**Table 8:** Plasma catalase activity (/g protein) across the groups

	Group A	Group B	Group C
Plasma catalase activity	0.3909 ± 0.029	0.2692 ± 0.031**	0.3858 ± 0.036
*P* (when compared with control)		0.0044	0.4133

** *P* < 0.01

**Table 9:** Tissue catalase activity (/g protein) across the groups

	Group A	Group B	Group C
Tissue CAT activity	0.3450 ± 0.027	0.2730 ± 0.043*	0.3858 ± 0.036
*P* (when compared with control)		0.0127	0.0651

* *P* < 0.05

Finally, there was no significant (*P* > 0.05) difference in both plasma and tissue MDA concentration of control and those of the animals pretreated with onion before cadmium administration. While animals treated with cadmium only shows a significant (*P* < 0.05) increase in both plasma and tissue MDA concentration [Tables 10 and 11, respectively]. Normally, decrease in MDA concentration is a sign of reduction in the activities of reactive oxygen species. It seems likely therefore that it was *Allium cepa* that reduced the oxidative stress that cadmium exposure could have caused in the onion pretreated animals.

**Table 10:** Plasma malondialdehyde concentration (micrograms/dl) across the groups

	Group A	Group B	Group C
Plasma MDA concentration	1496.79 ± 1.321	1679.48 ± 43.29*	1520.83 ± 13.26
*P* (when compared with control)		0.0113	0.2621

* *P* < 0.05

**Table 11:** Tissue malondialdehyde concentration (micrograms/g protein) across the three groups

	Group A	Group B	Group C
Tissue MDA concentration	1265.22 ± 2.285	1669.87 ± 14.61*	1244.8 ± 1.063
*P* (when compared with control)		0.0012	0.0558

* *P* < 0.05

It can be concluded that exposure to cadmium adversely affects renal functions (using renal clearance as a marker) due to cadmium's ability to cause oxidative stress by interfering with the activities of SOD and that of CAT, and thereby given freedom to free radicals [for example reactive oxygen species, (ROS)] to cause oxidation which manifests as increase in the concentration of MDA (in the case of lipid peroxidation). *Allium cepa*, however, prevents cadmium from adversely affecting renal functions. This would be a result of the anti-oxidant properties of *Allium cepa* which ensures reduced activity of ROS.
